# Genetic Diversity Analysis of 96 *Gossypium hirsutum*-*Gossypium barbadense* Introgression Lines and Early Maturing Northern China Cotton Lines Using a 40K Liquid-Phase Chip

**DOI:** 10.3390/genes17040388

**Published:** 2026-03-29

**Authors:** Pengpeng Chen, Yanlong Yang, Jiaxu Fang, Hang Yu, Yongmei Dong, Zengqiang Zhao, Yousheng Tian, Zongming Xie, Youzhong Li

**Affiliations:** 1 Key Laboratory of Cotton Biology and Genetic Breeding in the Northwest Inland Cotton Production Region, Ministry of Agriculture and Rural Affairs, Cotton Research Institute, Xinjiang Academy of Agricultural and Reclamation Science, Shihezi 832000, China; 2 Xinjiang Key Laboratory of Cotton Genetic Improvement and Intelligent Production, National Cotton Engineering Technology Research Center, Cotton Research Institute of Xinjiang Uyghur Autonomous Region Academy of Agricultural Sciences, Urumqi 830091, China; 3 College of Agriculture, Shihezi University, Shihezi 832000, China

**Keywords:** *Gossypium hirsutum-Gossypium barbadense*, SNP chip, genetic diversity, early maturity, nonsynonymous SNP, DCP1

## Abstract

**Background:** Genetic diversity and genetic differentiation between *Gossypium hirsutum*-*Gossypium barbadense* introgression lines (ILs) and early-maturing upland cotton lines are critical for resolving the core breeding contradiction in Xinjiang cotton region: narrow genetic basis of early-maturing cultivars and late maturity of ILs with superior fiber quality. Xinjiang is one of the major cotton-producing regions in China, and breeding high-quality early-maturing upland cotton adapted to local ecological conditions is essential for improving cotton yield and quality. However, the genetic relationship and differentiation between the two types of cotton germplasm remain unclear, which hinders the efficient utilization of germplasm resources in breeding. Therefore, this study aimed to clarify the genetic diversity and differentiation between the two germplasm types and identify key candidate loci related to early maturity and fiber quality, providing support for cotton breeding. **Results:** Here, we used a 40K Single Nucleotide Polymorphism chip to genotype core cotton germplasm in northern Xinjiang, and analyzed their population structure, genetic diversity and functional SNP loci associated with early maturity and fiber quality. The tested materials were clearly divided into two subgroups (ILs and early-maturing lines). Genetic diversity analysis revealed a significantly narrow genetic basis in the early-maturing subgroup, while the IL subgroup had higher genetic diversity. Specifically, the early-maturing subgroup showed lower nucleotide diversity and polymorphism information content compared with the IL subgroup, indicating that the genetic variation of early-maturing cotton germplasm in northern Xinjiang is relatively limited. A total of 25 non-synonymous SNPs were identified, among which the c.A613G:p.T205A mutation in *GH_D09G1484* (mRNA-decapping enzyme 1, DCP1) was a characteristic variation of early-maturing cotton, and a possible non-synonymous mutation in *GH_A09G2400* (Heat shock transcription factor A6b, HSFA6B) was associated with fiber development. These two candidate genes were annotated to be involved in plant growth and development, further supporting their potential roles in regulating cotton early maturity and fiber quality. **Conclusions:** This study clarified the genetic differentiation between the two types of germplasms and identified key candidate loci for early maturity and fiber quality, providing precise molecular markers and theoretical support for breeding high-quality early-maturing upland cotton adapted to Xinjiang’s ecological conditions. The results also highlight the value of *Gossypium hirsutum–Gossypium barbadense* introgression lines in enriching the genetic basis of early-maturing cotton, which can be further utilized to solve the core breeding contradiction in the Xinjiang cotton region.

## 1. Introduction

Upland cotton (*G. hirsutum* L.) is the primary source of natural textile fiber, yet in the Xinjiang region, the short frost-free season demands cultivars that combine early maturity with high fiber quality, which poses a critical challenge for local upland cotton breeding [1,2,3,4,5,6]. As the core cotton production base in China, Xinjiang boasts unique ecological advantages, including abundant light and heat resources, large diurnal temperature variations, and low incidence of diseases and insect pests, making it the primary guarantee for the stable supply of cotton in China [7,8,9]. With the continuous upgrading of demand for high-quality cotton in the textile industry and the stringent agronomic constraint of short frost-free period in the region, breeding new cotton lines with comprehensive traits of early maturity, high yield, and superior fiber quality has become the core objective of cotton genetic improvement in Xinjiang [10,11,12].

Sea island cotton (*G. barbadense* L.) is characterized by outstanding fiber properties, including long fiber length, high fiber strength and fine fiber fineness [13]. In contrast, upland cotton is highly favored for its high yield, strong adaptability and stable agronomic traits, and serves as the dominant cultivated species in global cotton production [14,15]. Sea island cotton–upland cotton introgression lines developed via interspecific hybridization and successive backcrossing integrate the superior fiber quality of sea island cotton into the genetic background of upland cotton, and have become vital germplasm resources for the fiber quality improvement of upland cotton [16]. However, existing sea island cotton introgression lines exhibit obvious shortcomings, including relatively late maturity, long growth period, and poor compatibility with the agronomic requirement for a short frost-free period in northern cotton-growing regions such as Xinjiang, which limit their large-scale popularization and application in local production [17]. By comparison, the early-maturing upland cotton lines widely cultivated in Xinjiang, after years of artificial selection, have evolved the traits of early flowering, early boll opening, and high pre-frost lint yield, and are highly adapted to the local ecological conditions [18,19,20]. However, these lines generally face problems such as a narrow genetic basis, a single genetic origin, and limited potential for the coordinated improvement of fiber quality and yield, which restricts the further improvement of cotton productivity in this region [21,22].

Traditional molecular marker technologies such as Restriction Fragment Length Polymorphism (RFLP), Random Amplified Polymorphic DNA (RAPD), and Simple Sequence Repeat (SSR) have been widely used in cotton germplasm diversity analysis. However, their low marker density, limited throughput, and insufficient polymorphism restrict their application in large-scale and high-precision cotton genetic research [23,24]. With the completion of sequencing and assembly of high-quality reference genomes of upland cotton and sea island cotton, SNP markers, due to their characteristics of wide distribution, high density, good stability, and ease of high-throughput detection in the genome, have become the most ideal molecular markers for crop genetic research [25]. Among them, high-throughput SNP chip technology has the advantages of high genotyping accuracy, fast detection speed, and low cost for large samples, and has been successfully applied in studies such as genetic diversity analysis of cotton, Quantitative Trait Loci (QTL) mapping of important agronomic traits, and molecular marker-assisted breeding, providing an efficient technical means for in-depth analysis of the genetic characteristics of cotton germplasm resources in Xinjiang [26,27]. However, the genome-wide genetic differentiation between these two germplasm types in Xinjiang remains uncharacterized, and the key nonsynonymous SNP loci regulating cotton early maturity and fiber quality have not been identified to date.

In this study, 96 cotton lines, including sea island cotton introgression lines widely planted in the Northern Xinjiang cotton region and local early-maturing upland cotton lines, were used as research materials. Genome-wide high-density genotyping was performed using the ZJU CottonSNP 40K liquid chip, developed by Shijiazhuang Boruidi Biotechnology Co., Ltd. (Shijiazhuang, China) and Zhejiang University (Hangzhou, China), which targets more than 40,000 high-quality SNPs covering approximately 25,000 genes and genic regions; the detailed design and validation of this chip were reported previously [28]. The aim of this study was to clarify the genome-wide SNP distribution characteristics, genetic diversity level and population genetic structure of the tested materials, reveal the genetic differentiation characteristics between sea island cotton introgression lines and early-maturing upland cotton lines, and mine key nonsynonymous mutation SNP loci related to important agronomic traits. The results of this study are expected to provide important genetic information and molecular markers for the genetic improvement of sea island cotton introgression lines, the breeding of new early-maturing and high-quality cotton varieties, and the efficient utilization of cotton germplasm resources in Xinjiang, laying a solid theoretical foundation for the sustainable development of cotton production in the northern cotton region. This study is expected to provide genetic insights and molecular markers for the genetic improvement of cotton germplasm in Xinjiang, and the findings will lay a theoretical foundation for the efficient utilization of sea island cotton introgression lines and the breeding of early-maturing and high-quality cotton varieties, thus supporting the sustainable development of cotton production in northern cotton-growing regions.

## 2. Materials and Methods

### 2.1. Materials

All tested cotton accessions are homozygous inbred lines with a stable genetic background, and the detailed information of these materials is provided in Supplementary Table S2. Young leaf samples were collected from a single representative plant per genotype. All plants were cultivated under standardized conditions: 28 °C, 16 h light/8 h dark photoperiod, with consistent light intensity, humidity, and water management. Genomic DNA was extracted using a modified CTAB method [29]. Briefly, leaf samples were ground in liquid nitrogen and incubated in 2% CTAB buffer containing 2% β-mercaptoethanol (Sangon Biotech, Shanghai, China) at 65 °C for 60 min. After extraction with chloroform-isoamyl alcohol (24:1) (Sangon Biotech, Shanghai, China) twice, DNA was precipitated with isopropanol (Sangon Biotech, Shanghai, China), washed twice with 75% ethanol (Sangon Biotech, Shanghai, China), and dissolved in TE buffer (Sangon Biotech, Shanghai, China).

### 2.2. Genotyping and SNP Analysis

Genotyping was performed using a 40K liquid-phase chip based on Genotyping by Targeted Sequencing (GBTS) [28] which was designed and developed by Zhejiang University, Hangzhou, Zhejiang, China and Breeder Biotechnology Co., Ltd., Shijiazhuang, Hebei, China. Genotyping was conducted by Breeder Biotechnology Co., Ltd., Shijiazhuang, Hebei, China. Clean reads after quality control were aligned using the BWA-MEM algorithm (v0.7.17) [30] with default parameters (Table S3). SNP loci with minor allele frequency (MAF) < 0.05, missing rate > 0.8, or identical to the TM-1 reference genome [31] were discarded. Finally, 40,844 high-quality SNP markers were identified for subsequent analyses.

### 2.3. SNP Variant Annotation

Based on the TM-1 reference genome and annotation file, genome-wide SNP variants were annotated and classified using ANNOVAR (v2.1.1) with default settings [32]. The variants were mainly divided into the following categories: SNPs located in intergenic regions, upstream regions, downstream regions, intronic regions, and exonic regions.

SNPs located in coding sequences were further classified into gain of stop codon, loss of stop codon, synonymous mutation, nonsynonymous mutation, frameshift deletion, and splice-site mutation.

### 2.4. Genetic Relationship Analysis

Based on the SNP variation information of the population, the phylogenetic tree was constructed using the neighbor-joining method in FastTree (v2.2) default parameters [33]. Population structure analysis was performed using ADMIXTURE (v1.3.0) with default parameters [34]. Both principal component analysis (PCA) and genetic relationship analysis were conducted using GCTA (v1.94.1) with default parameters and the -make-grm-alg option [35]. Quality control was first performed on the genotype data; samples and SNPs with a missing rate > 20% were removed, and the remaining missing values were imputed. Identity-by-state (IBS) values were calculated based on all quality-controlled SNPs without linkage disequilibrium filtering, and a robust genetic relationship matrix was then constructed from the IBS similarities to evaluate the pairwise genetic relationships among all experimental individuals.

### 2.5. Transcriptome Analysis

All publicly available RNA-seq data used in this study were retrieved from the CottonMD database [36]. Clean RNA-seq reads were aligned to the TM-1 reference genome [31] using HISAT2 (v2.2.2) [37] with default parameters. Gene expression levels were quantified using TPM (Transcripts Per Million) with StringTie [38] (v3.0.3, parameters: -fr-e-G) based on the high-quality mapped reads. TPM was used for standardization and comparison of gene expression levels across samples.

### 2.6. Download of Published Data

Illumina RNA-seq data and published cotton genome sequences of TM-1 were downloaded from the CottonMD database [36] (https://yanglab.hzau.edu.cn/CottonMD/, accessed on 3 July 2025).

## 3. Results

### 3.1. SNP Distribution Characteristics of Sea Island-Upland Cotton Introgression and Upland Cotton Populations

**Figure 1:**
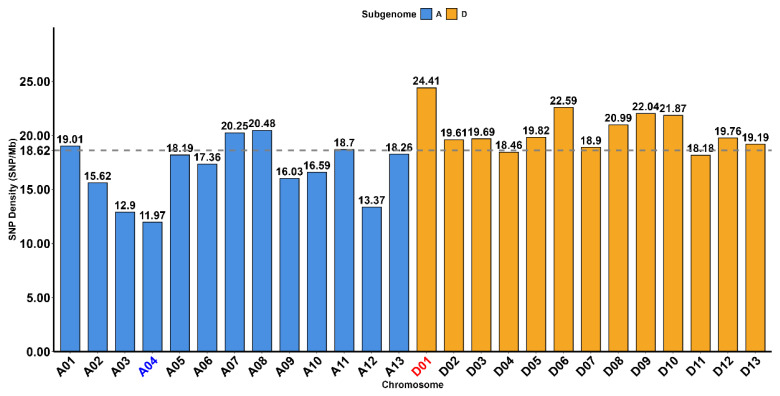
SNP density (SNPs/Mb) distribution across 13 chromosomes of the A and D subgenomes. The blue bars represent the A subgenome (chromosomes A01–A13), while the orange bars represent the D subgenome (chromosomes D01–D13). The dashed horizontal line indicates the overall average SNP density (18.62/Mb) across all chromosomes.

Ninety-six upland cotton lines were selected, including 80 ILs previously constructed by our research group and 16 cotton lines [2] long-term cultivated in Xinjiang (mainly early-to-medium maturing series lines). A total of 40,844 high-quality SNP markers were obtained, among which 24,123 SNP markers belonged to subgenome A and 16,717 SNP markers belonged to subgenome D. The number of SNPs on each chromosome ranged from 1050 (A04) to 2561 (A08) (Table 1). The average SNP density was 18.62/Mb, which was relatively evenly distributed on each chromosome; the maximum density was 24.41/Mb (D01, red), and the minimum density was 11.97/Mb (A04, blue) (Figure 1). These results indicate that the SNP markers obtained in this study are evenly distributed across the A and D subgenomes with a moderate density, providing a high-quality, high-resolution, reliable marker system for subsequent analysis of genetic diversity and population structure. With a set of high-quality, evenly distributed SNP markers successfully established for all tested cotton germplasm, we further conducted a comprehensive analysis of genetic diversity and population genetic structure of the 96 cotton lines to elucidate the genetic relationships and differentiation characteristics between sea island-upland cotton introgression lines and local early-maturing upland cotton lines in northern Xinjiang.

### 3.2. Genetic Diversity of ILs and Upland Cotton Lines

**Figure 2:**
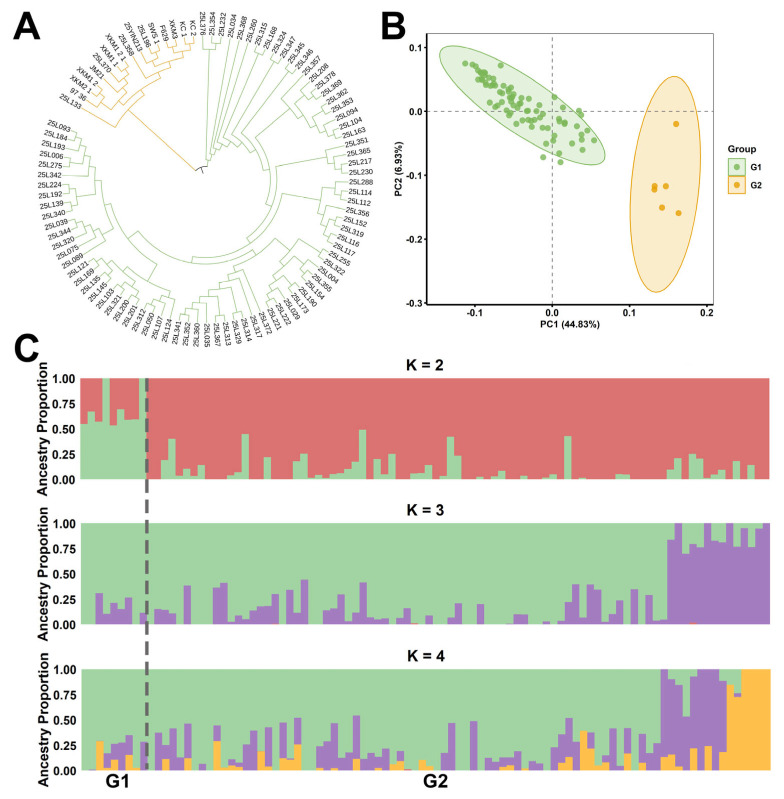
Clustering and population structure of sea-island introgression lines and early-maturing lines in upland cotton lines. (**A**) Phylogenetic tree of early maturing lines, different colors represent different groups (G1 and G2). (**B**) Principal component analysis. Different colors represent different groups. Arrows indicate important foreign-introduced parental lines. (**C**) Structure analysis with K = 2 and K = 3. The x-axis represents the different accessions. The orders and positions of accessions are consistent with those in the phylogenetic tree when K = 2. A vertical dashed line separates subgroup G1 and G2, consistent with the grouping in the phylogenetic tree.

To explore the genetic relationship among these materials, we calculated the genetic distances between the 96 materials and constructed a phylogenetic tree. The results confirmed that these materials were clearly divided into two subgroups (Figure 2A). Group I (G1) included 80 materials, mainly stable ILs cultivated through consecutive multiple generations, while Group II (G2) consisted of 16 materials, mainly the currently main promoted cultivated upland cotton lines in Xinjiang.

The results of Principal Component Analysis (PCA) and population structure analysis were consistent with those of the phylogenetic tree analysis (Figure 2B,C), confirming the accuracy of this classification. In this study, the centered Genetic Relationship Matrix (GRM) of the 96 materials was calculated based on the VanRaden algorithm. The results mean that the overall average centered relationship coefficient was −0.01, indicating that the overall genetic similarity of the population was slightly lower than the average level, and the genetic diversity was at a medium level (Table S4).

The average centered relationship coefficient within Group G1 was 0.02, suggesting that the genetic similarity among materials in this subgroup was only slightly higher than the population average, with relatively good genetic diversity. In contrast, the average centered relationship coefficient within Group G2 was as high as 0.39, which was significantly higher than those of Group G1 and the overall population. This revealed that the genetic similarity among materials in subgroup G2 was extremely high, with a highly narrow genetic basis and significantly lower genetic diversity than that in subgroup G1. These results indicate that the tested cotton germplasm can be clearly distinguished into two genetically distinct groups corresponding to *G. hirsutum*-*G. barbadense* introgression lines and local early-maturing upland cotton lines, which reflects the obvious genetic differentiation between the two germplasm types. Meanwhile, the significant difference in genetic diversity between the two groups further demonstrates that the local early-maturing upland cotton lines have a narrow genetic background, whereas the introgression lines maintain relatively abundant genetic variation, which provides valuable genetic resources for the genetic improvement of early-maturing upland cotton in Xinjiang. The identification of obvious genetic differentiation and distinct genetic diversity patterns between the two subgroups laid a foundation for further exploring the molecular genetic basis underlying their phenotypic differences in early maturity and fiber quality; thus, we next performed genome-wide SNP annotation and functional variant screening to identify key nonsynonymous SNP loci associated with these important agronomic traits.

### 3.3. Genetic Basis for the Improvement of ILs and Early-Maturing Upland Cotton

**Figure 3:**
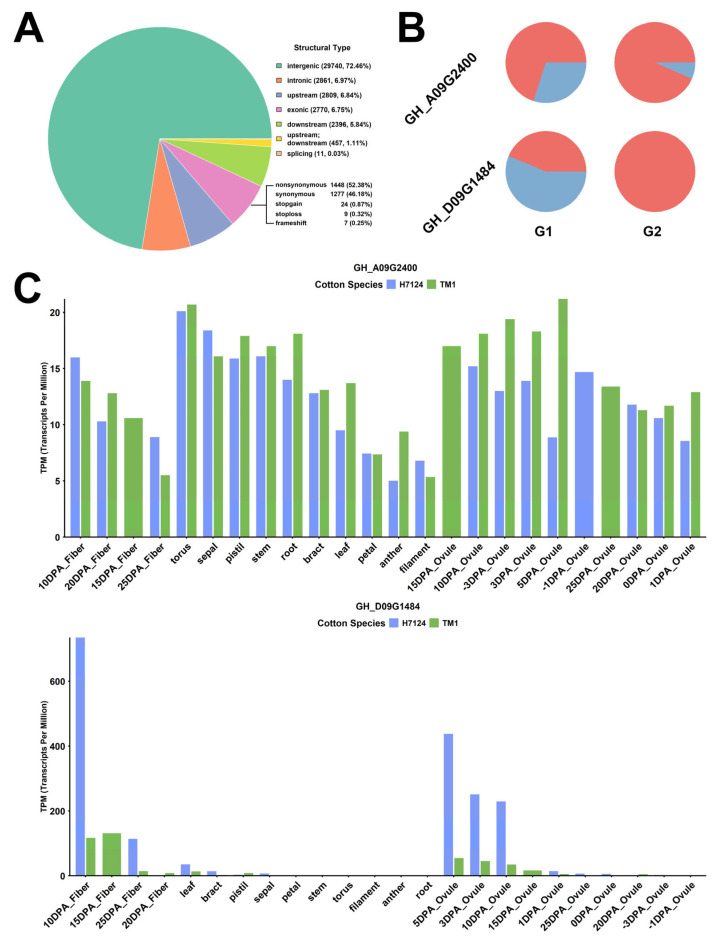
Genetic variation and expression profiles of *GH_D09G1484* and *GH_A09G2400*. (**A**) Statistics of SNP annotation information (**B**) Proportional distribution of SNPs occurring in *GH_D09G1484* and *GH_A09G2400* across different cotton populations. The pie chart shows the percentage of mutated SNPs (red) and unmutated SNPs (blue). (**C**) Expression profiles of *GH_D09G1484* and *GH_A09G2400* in various tissues.

Annotation of these SNPs showed that 29,740 SNP variants occurred in intergenic regions, 2770 SNPs in exon regions, 2861 SNPs in introns, 2809 SNPs in gene promoters, and 2396 SNPs in downstream regulatory regions (Figure 3A).

Filtering identified 25 nonsynonymous mutation SNPs (Table S1). These SNPs each had distinct characteristics in distribution frequency among different clusters; the SNPs carried by *GH_D09G1484* and *GH_A09G2400* had a higher distribution frequency in early-maturing lines (Figure 3B).

According to the transcriptome profiles (Figure 3C), *GH_D09G1484* showed stable expression in multiple cotton tissues, while *GH_A09G2400* was predominantly and highly expressed in ovules at 5 DPA and fiber tissues at 10 DPA and 15 DPA, indicating its crucial role in cotton fiber development.

**Figure 4:**
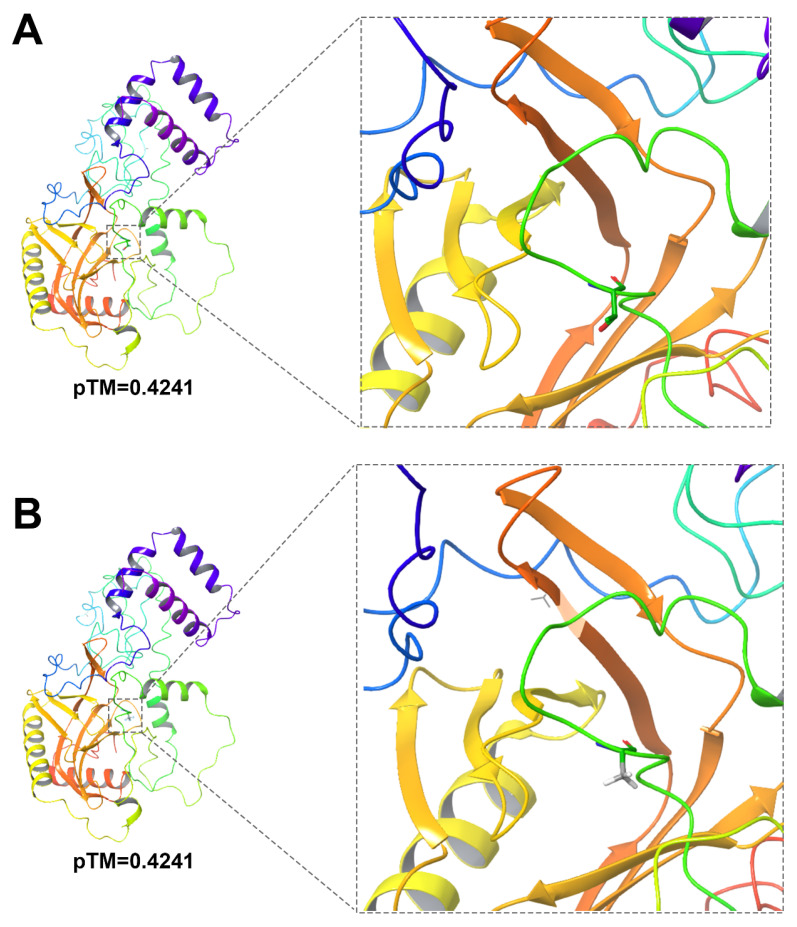
Structural schematic diagrams of the *GH_D09G1484* wild-type and T205A mutant. (**A**) Structure of the *GH_D09G1484* wild-type. (**B**) Structure of the T205A mutant of *GH_D09G1484*.

*GH_D09G1484* contained a nonsynonymous mutation in the 7th exon, where the 613th base in the coding region was substituted from adenine (A) to guanine (G), resulting in the substitution of threonine at the 205th amino acid of the protein with alanine (Figure 4). *GH_A09G2400* contained a nonsynonymous mutation in the 2nd exon (A to C), leading to the amino acid change from serine to arginine. It has been reported that heat shock transcription factors play a core role in regulating cotton fiber cell elongation, secondary wall synthesis, and stress responses during fiber development [39].

Here, a total of 25 nonsynonymous mutation SNP loci were identified, among which the mutation loci of *GH_D09G1484* and *GH_A09G2400* exhibited significantly higher distribution frequencies in early-maturing lines. Sequence analysis revealed an A-to-G base substitution in the seventh exon of *GH_D09G1484*, resulting in the replacement of threonine with alanine at the 205th amino acid residue of the encoded protein, while an A-to-C base substitution was detected in the second exon of *GH_A09G2400*, leading to a serine-to-arginine change in the corresponding protein. Notably, *GH_A09G2400* belongs to the heat shock transcription factor family, which has been previously demonstrated to be involved in the regulation of cotton fiber development. The distinct distribution patterns of these two genetic variants in early-maturing lines provide an important foundation for further investigating their putative functional association with key agronomic traits of early-maturing upland cotton in Xinjiang.

## 4. Discussion

The ZJU CottonSNP 40K chip enabled high-resolution genotyping of the tested germplasm, and the obtained SNP distribution characteristics provided a molecular basis for clarifying the genetic differentiation between ILs and early-maturing lines [8,28,40]. This genotyping strategy is particularly valuable for cotton breeding in northern Xinjiang, where ecological conditions impose strict agronomic constraints on early maturity and fiber quality.

Population structure analysis showed that ILs and early-maturing cotton lines were divided into two distinct groups, indicating that there were significant genomic differences between the two types of lines. The significant genetic differentiation between ILs and early-maturing cotton lines observed in this study is consistent with previous reports of obvious genomic divergence between sea island cotton introgression germplasm and local early-maturing upland cotton accessions [12,21]. Although the ILs cultivated in the early stage could be stably planted and cultured, they had large phenotypic differences, and their maturity time was slightly later [41] than that of early-maturing lines such as JM21 and 97-36. Referring to early-maturing lines, improving the existing ILs can provide a certain molecular breeding basis for cultivating early-maturing, long-staple and high-yield cotton. Based on the 40K liquid SNP chip technology, this study systematically analyzed the genetic diversity and genetic relationship of 16 main early-maturing upland cotton lines and 80 sea island-upland cotton introgression lines in Xinjiang cotton region. This suggested that the 96 cotton lines could be divided into two major genetic groups: the first group mainly consisted of ILs, and the second group mainly included early-maturing lines, among which the average kinship coefficient within the early-maturing line population was 0.39. SNP annotation results showed that only about 6.75% of the variations were located in gene coding regions or regulatory regions, which are important candidate regions mediating the observed phenotypic differences. Further screening of SNPs located in exons yielded a total of 25 nonsynonymous SNPs. The distribution frequency of these SNPs among different clustered populations showed a certain regularity, and this pattern was consistent with the characteristics of genetic differentiation between populations, which may result from various processes acting on the 25 identified nonsynonymous SNPs, such as genetic drift, direct or indirect selection, and local mutation. This indicated that, although the number of variants occurring in gene regions and regulatory regions was relatively lower than the genome-wide average, these loci were likely the primary causes of trait differences among different lines.

We also identified two functional genes, the decapping protein gene *GH_D09G1484* and the heat shock transcription factor gene *GH_A09G2400*, carrying nonsynonymous mutations associated with cotton earliness and fiber development, which may potentially contribute to the phenotypic variation in cotton lines in Xinjiang, providing important clues for subsequent functional gene mining and molecular marker-assisted breeding. The gene where this mutation occurred was annotated as decapping 1 (DCP1). As the core enzyme of the decapping pathway, DCP1 regulates the degradation efficiency of mRNA, affects the mRNA stability and translation efficiency of target genes genome-wide, and thereby regulates multiple growth and development processes of plants [39,42]. The gene *Gh_A09G2400* (HSFA6B) encodes heat shock transcription factor HSFA6B, which was highly expressed in sea island cotton cv. H7124 ovules at 5 DPA and fibers at 10 DPA and 15 DPA, suggesting that it may play a key role in fiber initiation and elongation. Notably, based on sequence homology, we speculate that this A subgenome gene likely represents the homeolog of GhHSFA6B-D, a transcription factor that was previously reported to maintain fiber yield under drought conditions in an ABA-independent manner by regulating GhIPS1-A, a homolog of myo-inositol 1-phosphate synthase [43]. Therefore, the variant loci of such transcription factors may be associated with the phenotypic variations related to fiber quality and earliness of cotton lines in Xinjiang.

High-efficiency genotyping was achieved using a high-density SNP chip, and the classification results were highly consistent with the subgroup classification reported previously, which further verified the differences between ILs and early-maturing lines. It has been confirmed in *Arabidopsis thaliana* and *Oryza sativa* that DCP1 is involved in processes such as seedling development, flowering time regulation, and stress responses (salt/drought/low temperature) [44,45]. Mutants often exhibit phenotypes including delayed flowering, retarded growth, and decreased stress resistance. DCP1 forms a decapping complex with DCP2, catalyzing the hydrolysis of the 5′-terminal m^7^GpppN cap structure of mRNA, initiating mRNA degradation or translation inhibition, and serving as a key node in post-transcriptional regulation [29,43,46]. The c.A613G:p.T205A mutation was fixed in all early-maturing upland cotton lines, suggesting that this mutation is likely a characteristic mutation of early-maturing upland cotton. We also acknowledge that the observed fixation of these SNPs in early-maturing lines may be influenced by population structure and SNP ascertainment bias; therefore, their functional roles should be interpreted cautiously. These results suggest that the identified nonsynonymous mutations in *GH_D09G1484* (DCP1) and *GH_A09G2400* (HSFA6B) are potential functional loci linked to early maturity and fiber development traits in cotton, respectively.

Subsequently, we predicted the three-dimensional (3D) structure of the Decapping 1 protein in early-maturing lines and ILs using AlphaFold3 (Figure 4A,B) [47]. We found that in the protein structure of ILs, the substitution of the polar and hydrophilic threonine with the non-polar, hydrophobic, and inactive alanine led to the loss of the polar hydrophilic hydroxyl group in the active loop region near the conserved domain, possibly resulting in a conformational change. Further validation of this mutation can be pursued through molecular dynamics simulations and experimental studies. This mutation putatively enhances the decapping function of GhDCP1, improves the binding efficiency to target mRNAs, accelerates the degradation of flowering repressor mRNAs, and thereby advances the flowering time. Although previous studies have been conducted on HSF family transcription factors [42], most of these studies focused on GhHSF6AB-D from the D subgenome, and there were problems of insufficient functional verification and incomplete analysis of regulatory mechanisms. The current work represents the first identification and functional characterization of GhHSFA6B on the A subgenome (*GH_A09G2400*), filling an important knowledge gap for this gene family in cotton. Future studies will further focus on the analysis of the fine regulatory mechanism of *GhHSF6AB* on cotton fiber yield and carry out systematic gene functional verification work. Therefore, the results of this study not only provide new gene targets and theoretical support for the molecular breeding of ILs, but also lay a foundation for the in-depth exploration and sustainable utilization of global cotton genetic resources.

Together, these results clarify the genetic differentiation and functional variations associated with cotton earliness and fiber quality. While the present findings offer useful insights, several limitations should be acknowledged. First, functional validation of the key candidate genes *GH_D09G1484* and *GH_A09G2400* was restricted to bioinformatic analysis; rigorous transgenic or gene-editing experiments are still needed to confirm their regulatory roles in cotton early maturity and fiber development. Second, the germplasm panel was mainly collected from northern Xinjiang; expanding the sampling range to other cotton-growing regions in China would help validate the universality of the identified loci. In future studies, we will conduct systematic functional verification and field trials to dissect the molecular regulatory networks of these genes and integrate them into marker-assisted breeding to accelerate the genetic improvement of cotton varieties adapted to local ecological conditions.

## 5. Conclusions

High-density genotyping using the ZJU CottonSNP 40K platform delineates population structure and genetic differentiation between upland-sea island cotton introgression lines and early-maturing upland cotton accessions in northern Xinjiang. Early-maturing cultivars display a narrowed genetic base, whereas introgression lines preserve elevated genetic diversity, indicating the value of interspecific introgression for broadening the genetic background of locally adapted cotton.

Functional variant screening identifies 25 nonsynonymous polymorphisms. The c.A613G:p.T205A mutation in *GH_D09G1484* (DCP1) is fixed in early-maturing accessions and may putatively enhance mRNA decapping to accelerate flowering. A nonsynonymous variant in *GH_A09G2400* (HSFA6B) is associated with fiber development and may contribute to enhanced fiber quality. These loci constitute robust molecular targets for the synchronous improvement of earliness and fiber traits. Characterization of genomic divergence and key functional polymorphisms establishes a foundational framework for precision molecular breeding in Xinjiang cotton.

Limitations include the lack of experimental validation for candidate gene function via transgenic or gene-editing assays, as well as the restriction of germplasm to northern Xinjiang environments; broader sampling across ecological zones will be required to validate the universality of the identified loci.

In marker-assisted breeding, the diagnostic SNP for earliness enables efficient selection of early-maturing individuals in segregating populations, while the fiber-linked polymorphism supports pyramiding of favorable alleles to upgrade fiber performance. Collectively, these findings enable the accelerated development of superior early-maturing, high-quality cotton cultivars adapted to the short frost-free period in northern cotton-growing regions and reinforce the genetic foundation for sustainable cotton production in Xinjiang.

## Figures and Tables

**Table 1:** Distribution of SNPs across different chromosomes (Chr).

Chr	Length (bp)	SNP Number	Chr	Length (bp)	SNP Number
A01	118,174,371	2247	D01	64,698,102	1579
A02	108,272,889	1691	D02	69,777,850	1368
A03	111,586,618	1439	D03	53,896,199	1061
A04	87,703,368	1050	D04	56,935,404	1051
A05	110,845,161	2016	D05	63,929,679	1267
A06	126,488,190	2196	D06	65,459,843	1479
A07	96,598,283	1956	D07	58,417,686	1104
A08	125,056,055	2561	D08	69,080,421	1450
A09	83,216,487	1334	D09	52,000,373	1146
A10	115,096,118	1910	D10	66,881,427	1463
A11	121,376,521	2270	D11	71,358,197	1297
A12	107,588,319	1438	D12	61,693,100	1219
A13	110,367,549	2015	D13	64,447,585	1237

## Data Availability

The datasets presented in this study can be found in publicly available database.

## Supplementary Materials

The following supporting information can be downloaded at: https://www.mdpi.com/article/10.3390/genes17040388/s1. Table S1: Information of selected SNPs; Table S2: Summary of test material information; Table S3: Sequencing statistics of the 96 cotton accessions; Table S4: Average kinship coefficients for the total population and G1/G2 subgroups.
